# Optical Quality and Centration Stability of a Novel Capsule Reconstructor for Intraocular Lens Fixation: Laboratory Evaluation of the fixOflex Device

**DOI:** 10.1167/tvst.15.7.5

**Published:** 2026-07-06

**Authors:** Leoni Britz, Maximilian Hammer, Grzegorz Łabuz, Zhiyi Wu, Ioannis George Pallikaris, Gerd Uwe Auffarth

**Affiliations:** 1 The David J. Apple Laboratory for Vision Research, Heidelberg, Germany; 2 Department of Ophthalmology, University Hospital Heidelberg, Heidelberg, Germany; 3 Faculty of Biosciences, Heidelberg University, Heidelberg, Germany; 4 Department of Ophthalmology, Zhejiang University, Hangzhou, China; 5 Institute of Vision and Optics, Medical School, University of Crete, Heraklion, Greece

**Keywords:** intraocular lenses (IOLs), optical bench testing, capsular bag stabilization, modulation transfer function, straylight

## Abstract

**Purpose:**

To evaluate the in vitro optical quality of intraocular lenses (IOLs) implanted into the novel fixOflex device, a capsular bag stabilization concept.

**Methods:**

Five monofocal (AcrySof IQ SN60WF) and five trifocal (AcrySof IQ PanOptix) IOLs of the same dioptric power were assessed in a laboratory setting before and after implantation into five fixOflex devices. Optical quality was analyzed using modulation transfer function (MTF) and forward light scatter (straylight) across clinically relevant aperture sizes and spatial frequencies. Vertical and horizontal IOL decentration within the device was also quantified.

**Results:**

Implantation into the fixOflex preserved the high optical quality of the IOLs. Changes in MTF were minimal and not statistically significant (*P* > 0.1) across all focus distances, spatial frequencies, and aperture sizes. The fixation clips caused a slight increase in straylight (0.10 log(s) at a 5.5-mm pupil), which remained well below clinically relevant thresholds. The device provided high IOL centration, with decentration below 0.03 mm along the clip axis and below 0.25 mm along the haptic axis, without significant tilt.

**Conclusions:**

Implantation of monofocal and multifocal IOLs into the fixOflex device preserved their high optical quality and provided stable centration under controlled laboratory conditions.

**Translational Relevance:**

These findings provide a translational basis for considering the fixOflex as a stabilizing platform for decentration-sensitive IOL designs, supporting clinical evaluation in patient populations.

## Introduction

In recent decades, cataract surgery has undergone substantial advancements, driven by the introduction of new intraocular lens (IOL) designs,^1^ enhanced surgical techniques,^2^^,^^3^ and the development of advanced surgical devices.^4^ However, several challenges persist, which continue to compromise both visual and functional outcomes. Posterior capsule opacification (PCO) remains the most common condition affecting visual quality, developing on average in over 30% of patients within 5 years postoperatively.^5^ PCO results from the migration of residual lens epithelial cells onto the central posterior capsule,^6^ whereas fibrosis and shrinkage of the anterior capsule can lead to significant capsule phimosis in 1.40% of the patients.^7^ In both instances, capsular fibrosis can substantially impair visual function by reducing visual acuity and contrast sensitivity or by increasing forward light scattering, leading to glare symptoms.^8^ Another major complication associated with capsular shrinkage is IOL decentration, which can particularly affect the optical performance of aspheric, toric, or multifocal intraocular lenses.^9^^–^^13^

**Figure 1.**
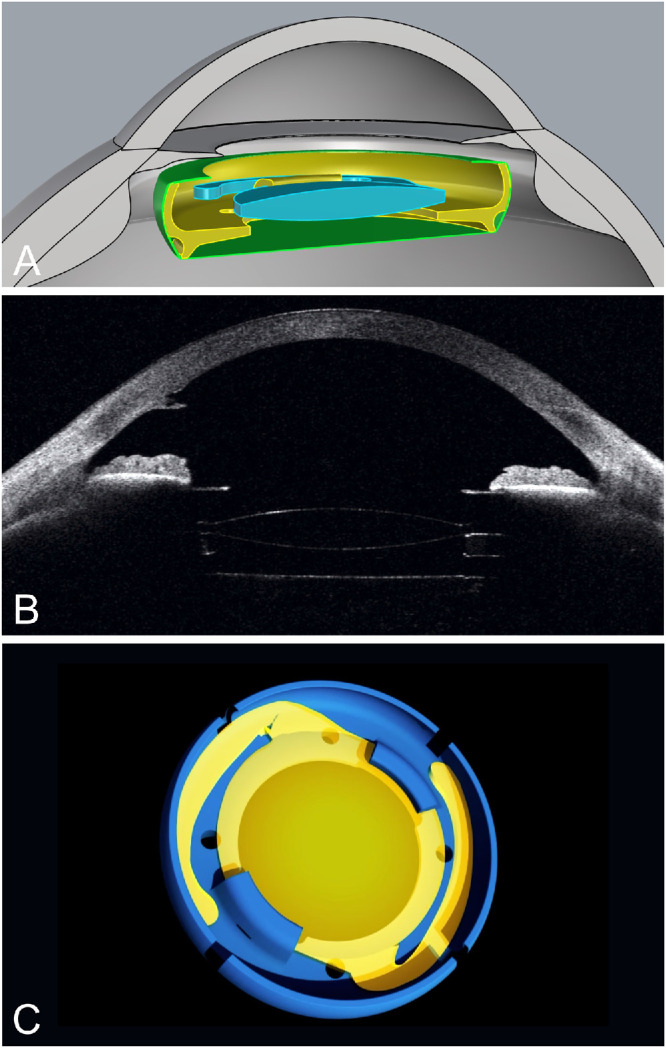
Schematic cross-sectional view (**A**) illustrating the principle of mechanical expansion of the capsular bag (*green*) by the fixOflex device (*yellow*) and the positioning of a one-piece C-loop IOL (*blue*) within the device. Optical coherence tomography (OCT) image (**B**) showing effective expansion of the capsular bag by the fixOflex device 1 month postoperatively in a patient's eye, provided for illustrative purposes only and not part of the present in vitro study, with stable, well-centered positioning of a one-piece C-loop IOL within the device and no contact with the capsular bag. Schematic top view (**C**) illustrating the positioning of a one-piece C-loop IOL within the fixOflex device.

The fixOflex device is a novel intraocular implant designed as a capsule reconstructor to mechanically stabilize the capsular bag and prevent postoperative complications associated with capsular fibrosis, shrinkage, and therefore IOL dislocation.^14^^,^^15^ It is a circular ring-shaped device made of hydrophilic acrylic with a sharp-edge profile, measuring 9.8 mm in diameter and 1.84 mm in height (Fig. 1). The device is inserted into the capsular bag during cataract surgery, in addition to the IOL, and acts by reexpanding the capsular bag, thereby restoring a more physiological contour after removal of the crystalline lens.^16^ This expansion is intended to counteract fibrotic contraction processes that may otherwise lead to capsular shrinkage and IOL misalignment. A central opening within the fixOflex accommodates standard C-loop haptic IOLs with a 6.0-mm optic diameter, which are held in place by opposing fixation clips. This combination of capsular expansion and mechanical fixation is intended to support stable IOL positioning with regard to both centration and tilt.

The present study is the first to provide a quantitative optical quality assessment of this novel implant. Under standardized and controlled laboratory conditions, we evaluated modulation transfer function (MTF)–based image quality, horizontal and vertical IOL decentration, and glare potential through straylight measurements. While clinical investigations of the fixOflex implant are currently underway, the aim of this preclinical study was to determine whether the implantation of aspheric monofocal and trifocal IOLs into the device preserves their expected optical performance characteristics.

## Methods

### Light Microscopy

To evaluate potential straylight sources of the IOLs implanted into the fixOflex device (EyePCR B.V., Heraklion, Greece), images of the implants at a 3-mm and 5.5-mm aperture were taken using a surgical microscope setup with a camera (Blackmagic Pocket Cinema Camera 4K; Blackmagic Design Pty Ltd, Melbourne, Australia). All measurements were conducted with the implants maintained in a hydrated state.

### Straylight Measurements

Straylight was assessed in a laboratory setting under several conditions: caused by five fixOflex implants alone; for five monofocal, hydrophobic, aspheric IOLs of identical design and dioptric power (+20 diopters; Acrysof IQ SN60WF; Alcon, Geneva, Switzerland); and for those five IOLs implanted into five different fixOflex devices. Straylight was measured using the C-Quant straylight meter (Oculus Optikgeräte GmbH, Wetzlar, Germany) and a standardized setup in our laboratory.^17^^–^^21^ Measurements were conducted using apertures of 3 mm and 5.5 mm, simulating pupil sizes at photopic and scotopic conditions, respectively. A customized adapter containing an IOL holder within a glass cuvette filled with balanced saline solution was used to position the implant in front of the device. The C-Quant test field was projected onto the implant through a plano-convex lens. A diaphragm blocked the scattering source, allowing only the light scattered by the implant to be perceived by the examiner. Straylight measurements were performed twice for each implant and aperture, and the mean value was calculated. Before testing, the straylight of the optical setup without a fixOflex device or IOL in place was measured twice, and the mean value was subtracted from all subsequent readings. All measurements were conducted by a single examiner (L.B.), who was blinded to both the aperture size and the implant under investigation. The logarithmic straylight data are presented as median [interquartile range]. The C-Quant is a well-validated method for measuring straylight, with high reproducibility and repeatability demonstrated in both in vivo and in vitro settings.^22^^–^^24^

### Modulation Transfer Function

The MTF served as an additional optical quality parameter, which was evaluated with a previously described, well-established setup.^25^^–^^27^ An optical bench system (Optispheric IOL Pro 2; Trioptics GmbH, Wedel, Germany) featuring monochromatic light (546 nm) and standard apertures of 3.0 mm and 4.5 mm was used, simulating typical pupil sizes under photopic and mesopic conditions relevant for visual performance, respectively. The MTF was assessed for the distance focus for five monofocal IOLs (+20 diopters; Acrysof IQ SN60WF; Alcon) and for the near, intermediate, and distance focus for five trifocal IOLs (+20 diopters; AcrySof IQ PanOptix Trifocal IOLs; Alcon), both independently and following implantation into the fixOflex devices.

All IOLs shared the same optical power, material, design, and manufacturer to isolate the effect of the fixOflex device. Particular care was taken to ensure consistent and reliable positioning of each IOL within the fixOflex device and the model eye. Using predefined reference marks on the model eye, identical vertical haptic orientation and IOL centration were ensured for every assessment. Each measurement was automatically repeated three times by the optical bench, and the means were used for analysis. MTF values were obtained for the sagittal and tangential meridians of the IOLs at spatial frequencies of 50- and 100-line pairs per millimeter (lp/mm). A model cornea with a physiological spherical aberration of +0.20 µm was used in this study to simulate human corneal optics and ensure clinically relevant MTF measurements. All measurements were conducted by a single investigator (Z.W.), who was blinded to the sample. The MTF is a well-validated parameter, allowing objective evaluation of the in vitro optical performance of IOLs.^25^^,^^28^

### Decentration Measurement

The previously described light microscopy setup equipped with a camera was used to document the centration of the five trifocal IOLs (+20 diopters; AcrySof IQ PanOptix Trifocal IOLs; Alcon) placed into the five different fixOflex devices. Relative decentration was assessed by determining the optic centers of both the fixOflex and the IOL and comparing their horizontal and vertical deviations using image analysis in Fiji (version 2.14.0/1.54f; ImageJ; National Institutes of Health, Bethesda, MD, USA). In addition, the microscopy setup was used to visually assess for signs of IOL tilt, although tilt was not measured quantitatively.

### Statistical Analysis

Statistical analysis was carried out using Prism 10.2.2 (GraphPad Software, La Jolla, CA, USA). The Kolmogorov–Smirnov test was used to assess normality. Paired Student's *t*-test was performed to evaluate statistical significance.

This in vitro study did not involve human participants or animals and therefore did not require ethical approval.

## Results

### Straylight Measurements

**Figure 2.**
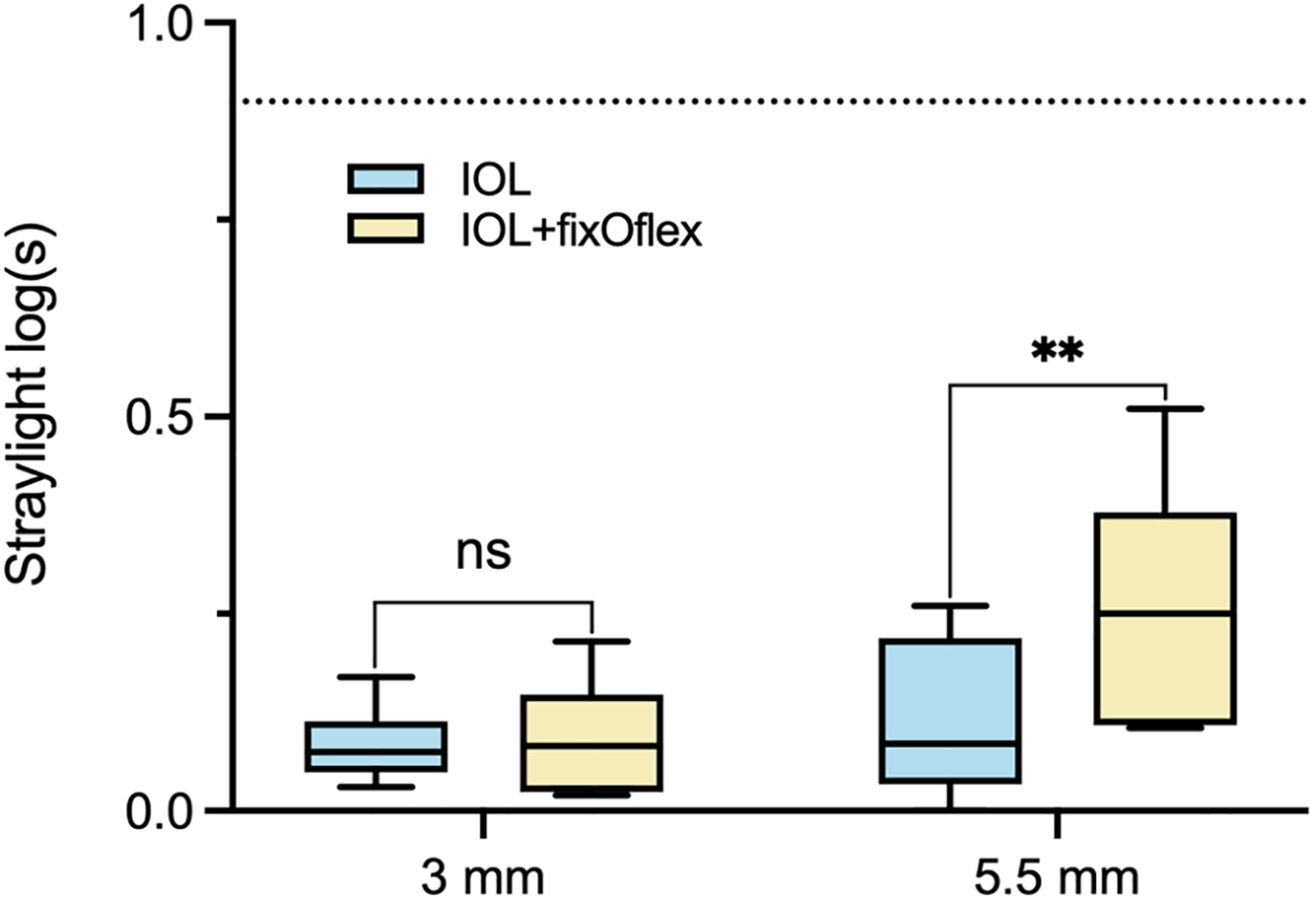
Straylight levels for the monofocal IOLs alone and when implanted into the fixOflex device at 3-mm and 5.5-mm aperture sizes. Implantation of an IOL into a fixOflex caused a statistically significant (*P* = 0.0091) increase of +0.10 [0.11] log(s) in straylight at an aperture of 5.5 mm versus the IOL alone. These values were still far below the straylight of a crystalline, young lens [*dotted line*, 0.9 log(s)].

The fixOflex device alone generated a straylight value of 0.26 [0.08] log(s) when measured with the 5.5-mm aperture, while no additional straylight could be measured with the 3-mm aperture. The five monofocal IOLs alone demonstrated a median straylight of 0.07 [0.04] log(s) for the 3-mm aperture and 0.08 [0.12] log(s) for the 5.5-mm aperture (Fig. 2). The additional straylight caused by the fixOflex device at larger apertures was reproducible but smaller when measured with IOLs implanted into the device. A statistically significant (*P* = 0.0091) increase in straylight of 0.10 [0.11] log(s) was measured for IOLs implanted into the devices at a 5.5-mm aperture compared to the IOLs alone. No statistically significant difference was found for the 3-mm aperture (*P* > 0.5) when comparing these two groups. It is important to note that, although the average increase in straylight reached statistical significance, it remains well below the level observed clinically. To facilitate evaluation of the absolute straylight values, the straylight of a young, healthy eye, which is 0.9 log(s), is given as a reference.

**Figure 3.**
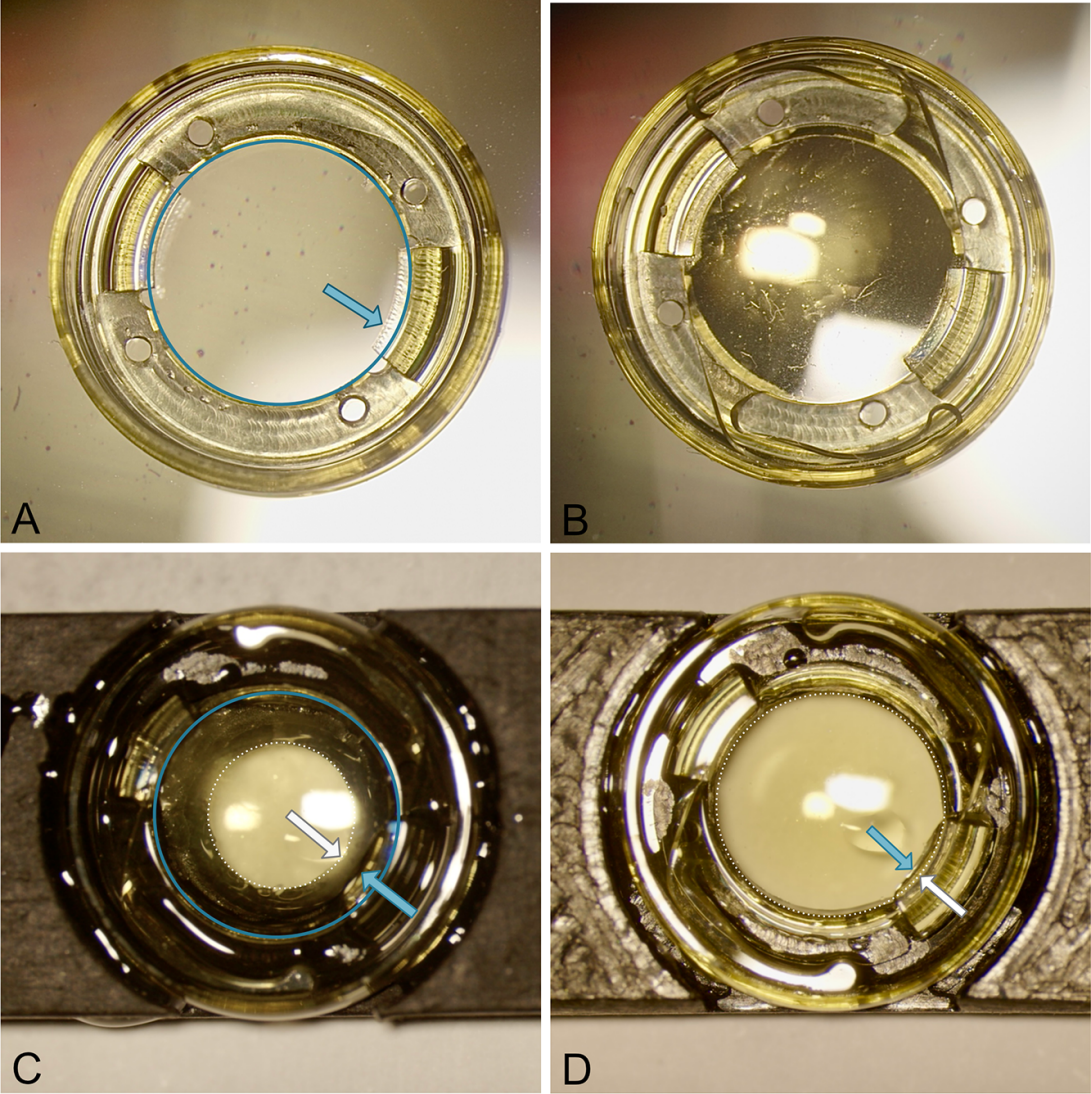
The fixOflex is designed with a central opening (*blue circle*, **A**) that is slightly smaller than the 6-mm optic of a standard C-loop haptic IOL to ensure its secure positioning (**B**). Additionally, the device includes two opposing clips for IOL fixation (*blue arrows*, **A**, **C**, **D**), which slightly encroach into the optical axis at larger pupil sizes, as simulated by a 5.5-mm aperture in our setup (*white dotted circle*, **D**). In contrast, no such protrusion is observed with a 3-mm aperture (*yellow dotted circle*, **C**), resulting in no additional straylight at smaller pupil diameters.

To investigate the source of increased straylight when IOLs were implanted into the fixOflex, we captured overview images of the implants (Fig. 3). The images revealed that the device clips securing the IOL protruded slightly into the optical axis at a 5.5-mm aperture, leading to the generation of additional straylight. In contrast, no such protrusion was observed at a 3-mm aperture, and consequently, no additional straylight was detected under these conditions.

### Modulation Transfer Function of Monofocal IOLs

**Figure 4.**
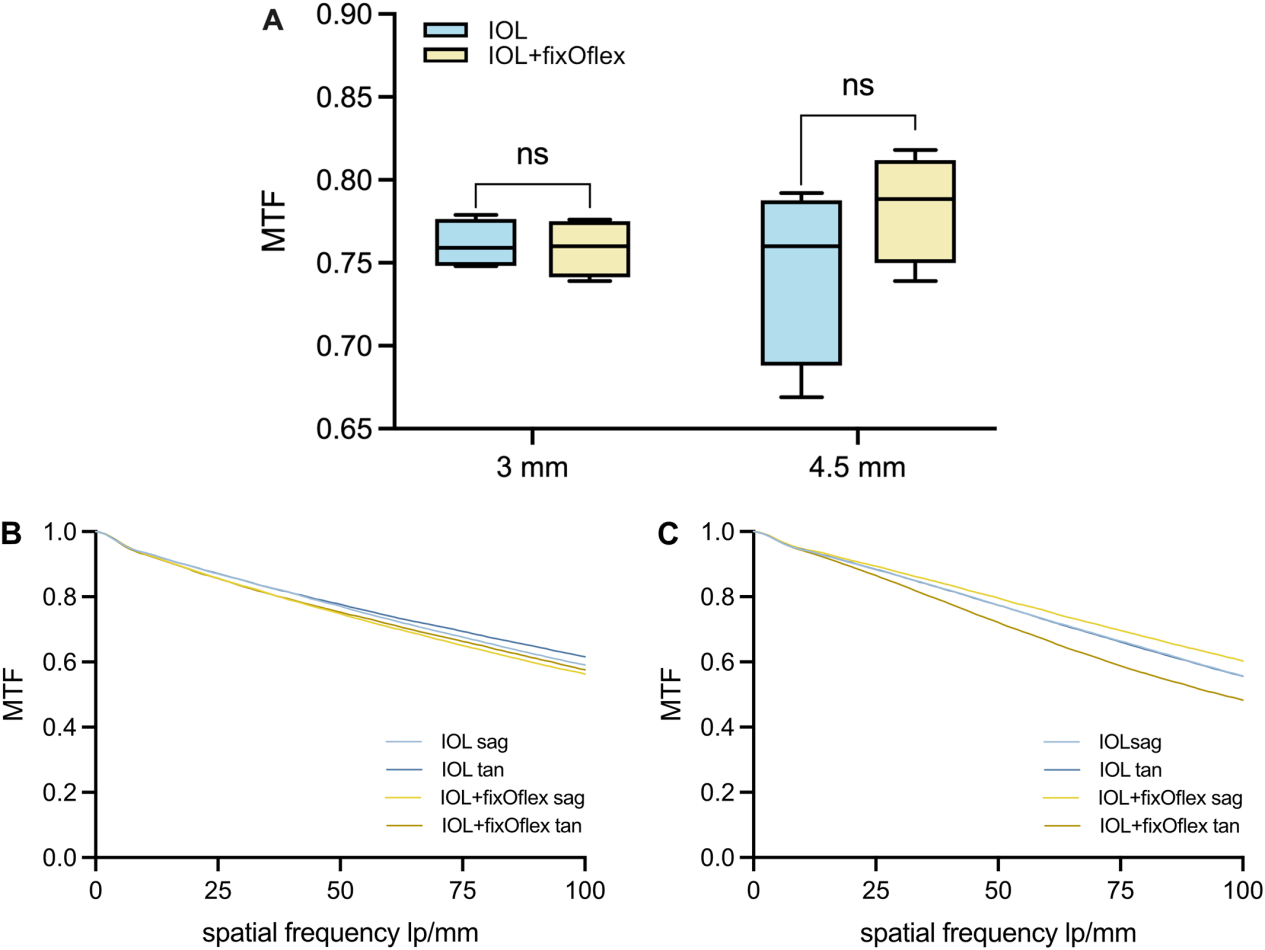
Optical quality assessment of the monofocal Acrysof SN60WF IOLs prior to and after implantation into the fixOflex of different spatial frequencies and aperture sizes. The IOLs demonstrated excellent optical quality at the distance focus, with a value higher than 0.67 at 50 lp/mm for both aperture sizes (**A**). When the IOLs were positioned in the device, minimal, not statistically significant (*P* > 0.2) changes in MTF were observed (**A**). No significant changes in MTF were detected across other spatial frequencies prior to versus after implantation into the devices, for both 3-mm (**B**) and 4.5-mm aperture sizes (**C**) and for the sagittal (sag) and tangential (tag) IOL meridian.

The five monofocal aspheric AcrySof IQ SN60WF IOLs demonstrated consistently high optical image quality across both aperture sizes, and implantation into the fixOflex did not result in any relevant reduction in MTF performance (Fig. 4). No significant differences (*P* > 0.05) were found between the sagittal and tangential meridians, and the data were therefore grouped. At 50 lp/mm, the mean MTF at the 3-mm aperture was 0.76 ± 0.01 for the IOLs alone, with a small decrease of −0.013 ± 0.01 after placement into the fixOflex. At the 4.5-mm aperture, the corresponding values were 0.75 ± 0.05 and +0.02 ± 0.04, respectively (*P* > 0.2 and *P* > 0.65).

Similarly, high MTF values were observed at 100 lp/mm, measuring 0.581 ± 0.01 at the 3-mm aperture and 0.54 ± 0.07 at the 4.5-mm aperture. When positioned in the fixOflex devices, the changes at this higher spatial frequency were minimal and not statistically significant (*P* > 0.1), with a slight increase of +0.001 ± 0.009 at 3 mm and a decrease of −0.02 ± 0.004 at 4.5 mm.

### Modulation Transfer Function of Trifocal IOLs

The optical quality of the trifocal PanOptix IOLs was evaluated analogously to the monofocal lenses, at 3-mm and 4.5-mm apertures, for both sagittal and tangential meridians, and at spatial frequencies of 50 and 100 lp/mm. As no relevant differences between meridians were observed (<0.05 ± 0.03; *P* > 0.1), the data were averaged. Measurements were conducted for the far, intermediate, and near foci.

**Figure 5.**
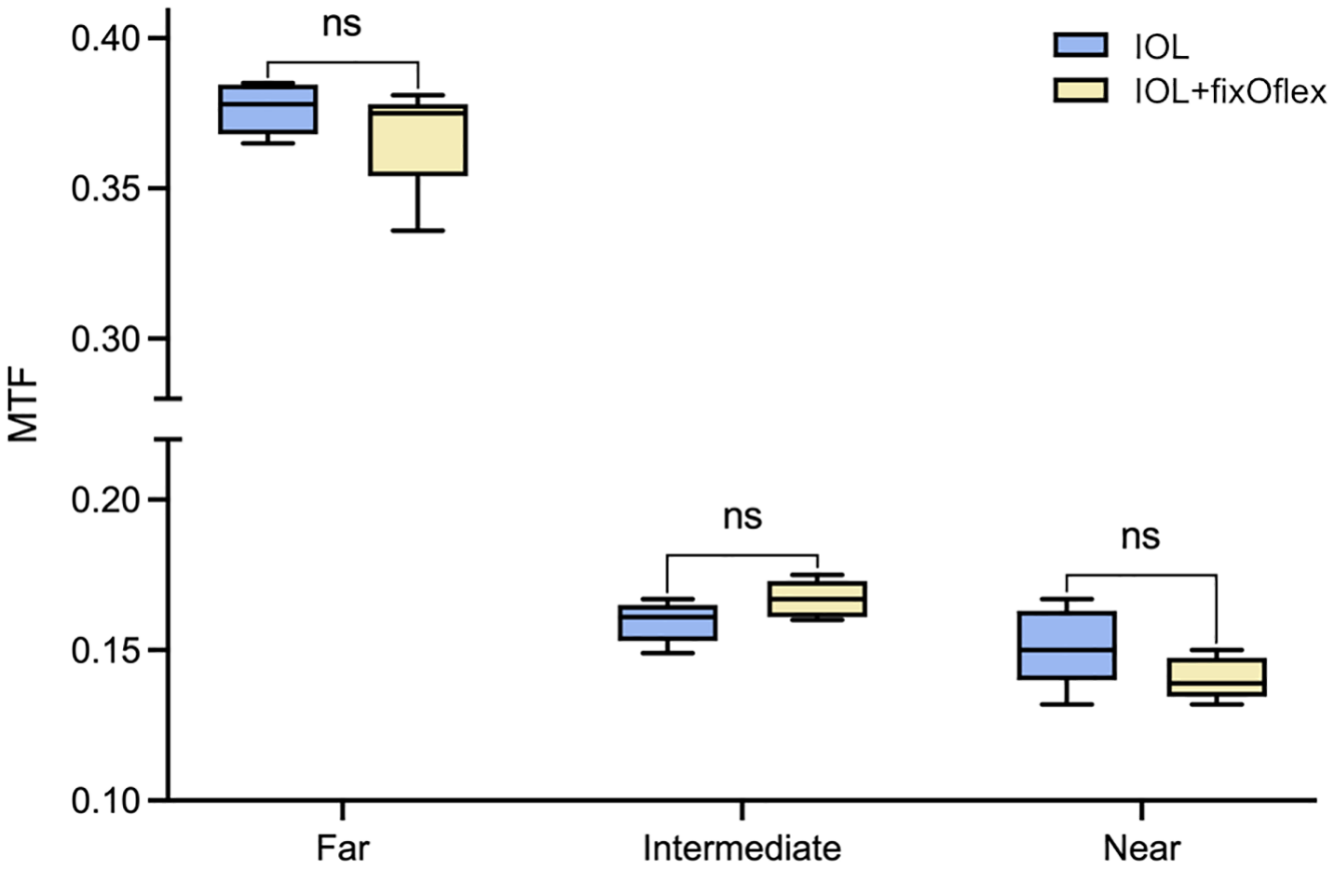
Optical quality of trifocal IOLs measured using the MTF at a 3-mm aperture and 50 lp/mm. MTF values were assessed for far, intermediate, and near foci, both for the IOLs alone and when positioned in the fixOflex. The PanOptix IOL demonstrated consistently high optical quality across all focal points, with no significant changes observed when the IOLs were placed in the device.

At 50 lp/mm and a 3-mm pupil, the PanOptix lenses demonstrated high optical performance, with MTF values of 0.37 ± 0.007 (far), 0.16 ± 0.006 (intermediate), and 0.15 ± 0.009 (near). Implantation into the fixOflex resulted in only minimal, nonsignificant changes (−0.01, +0.02, −0.01; *P* > 0.1) across the three foci (Fig. 5). For the 4.5-mm aperture, the corresponding MTF values were 0.32 ± 0.03, 0.14 ± 0.02, and 0.14 ± 0.01, with similarly small and nonsignificant changes of ≤0.04 (*P* > 0.6). At the higher spatial frequency of 100 lp/mm, statistically significant reductions were detected for the intermediate focus at 4.5 mm and the near focus at both pupil sizes (*P* < 0.05). However, these differences were minimal, with absolute reductions <0.06 in the MTF.

### Centration of Trifocal IOLs in the fixOflex

**Figure 6.**
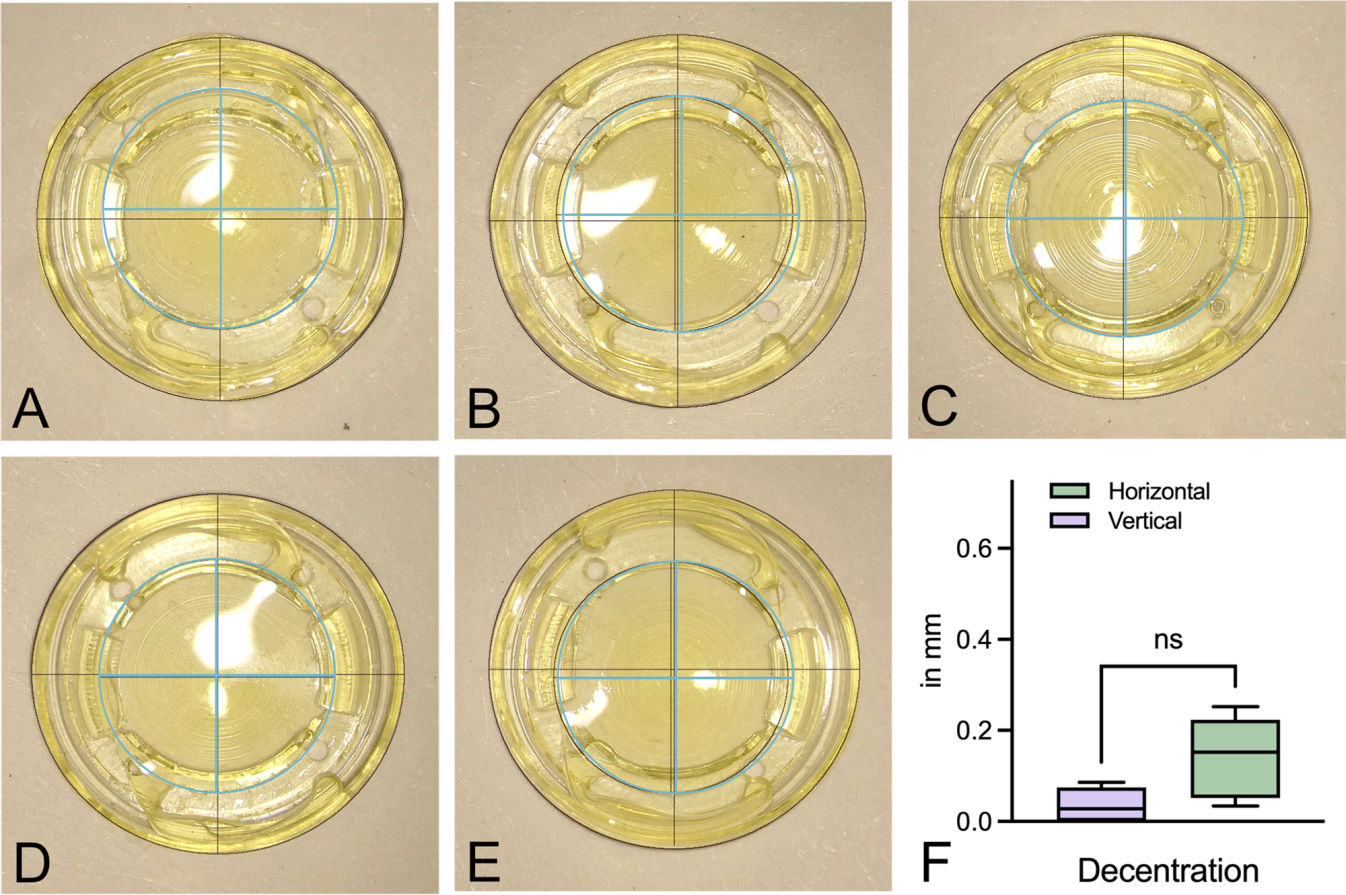
Evaluation of IOL decentration in the fixOflex devices (**A**–**E**) demonstrated high centration, with maximal decentration lower than 0.25 mm and higher, but no significant difference (*P* > 0.06) for the horizontal versus the vertical axes (**F**). The *blue circle* represents the optic of the IOL, while the *black circle* denotes the outer diameter of the fixOflex (**A**–**E**).

We further evaluated the centration of the IOLs within the fixOflex by measuring both horizontal and vertical decentration (Fig. 6). Overall, the IOLs exhibited minimal decentration, with a vertical decentration of 0.03 ± 0.04 mm and a horizontal decentration of 0.25 ± 0.09 mm. Although decentration along the horizontal axis was higher, there was no statistically significant difference in the likelihood of decentration between the two axes (*P* > 0.06).

## Discussion

This study represents the first investigation into the optical quality of IOLs implanted into the novel fixOflex device, which was developed to mitigate common postoperative complications in cataract surgery. Our findings show that monofocal and multifocal IOLs maintained high optical quality when implanted into the fixOflex, with no significant differences compared to isolated IOL measurements. Implantation into the device resulted in stable IOL centration, and the glare potential from straylight remained negligible.

We evaluated straylight at clinically relevant pupil sizes, using 3 mm to simulate photopic conditions and 5.5 mm to simulate scotopic conditions or the physiologically larger pupil diameters in younger patients. Assessing straylight at larger pupil sizes is clinically important, as scatter increases with pupil dilation, and glare-related symptoms predominantly occur under low-light conditions.^29^ We observed that the fixation clips of the fixOflex led to a minimal but statistically significant increase of 0.10 log(s) in straylight at the 5.5-mm pupil size, whereas no significant change was detected at the 3-mm aperture.

An increase of 0.3 log(s) corresponds to a doubling of ocular straylight and may cause noticeable visual impairment.^30^ The 0.10-log(s) increase induced by the fixOflex clips remained far below this threshold and well below the straylight level of a healthy young eye (∼0.9 log(s)).^30^^,^^31^ Thus, a clinically meaningful effect on visual performance is unlikely. In addition, a 5.5-mm aperture corresponds to an even larger effective pupil diameter in vivo, further reducing the relevance of the clips as a potential glare source.

The evaluation of straylight across different intraocular implants is critical when assessing overall optical quality.^19^^,^^32^^–^^35^ While visual acuity remains a key metric, elevated levels of straylight can significantly reduce patient satisfaction, even when visual acuity is excellent.^17^^,^^30^^,^^36^ Because the fixOflex is designed not only for standard cataract surgery but also for procedures such as refractive lens exchange and pediatric cataract surgery, maintaining low straylight levels is essential to ensure high patient satisfaction and optimal visual performance.

We evaluated the MTF of monofocal and multifocal IOLs before and after implantation into the fixOflex under standardized laboratory conditions. Both lenses maintained high optical quality, with no clinically relevant changes. For monofocal IOLs, MTF differences were ≤0.02 under all conditions; for multifocal IOLs, differences were ≤0.04 at 50 lp/mm and ≤0.06 at 100 lp/mm. These values fall well within the ISO 11979-2 reproducibility limits, indicating measurement variability rather than true optical differences. No differences between the sagittal and tangential meridians were observed, further indicating that the fixation clips do not influence the optical image quality. Consequently, the optical quality of the evaluated IOLs remains effectively unchanged when fixated within the device, as visualized by the United States Air Force (USAF) target chart (Supplementary Fig. S1).

A factor that can significantly influence optical quality is IOL decentration or tilt, which is particularly critical for aspherical and multifocal IOLs.^9^^,^^13^ We evaluated IOL centration within the fixOflex device and found a low potential for decentration, with a slightly—but not significantly—higher risk along the C-loop haptic axis. Along this axis, the IOL is supported by its haptics clamped into the device's ring (Figs. 1, 6). The flexibility of the C-loop haptics likely allows for greater variability in the final IOL position within the fixOflex device, resulting in a higher degree of decentration along the horizontal axis compared to the vertical axis. The clips along the orthogonal, vertical axis provide opposing fixation of the IOL's optic, which likely provides greater stability and results in more consistent centration along the vertical axis. Positioning the IOL with the haptics perpendicular to the clips allows for the greatest degree of decentration; alternative positioning may reduce it. Whether this minor asymmetry in IOL centration within the device affects in vivo positioning, including the rotational alignment of toric IOLs, should be evaluated in future clinical studies.

In clinical settings, average IOL decentration ranges from 0.20 to 0.70 mm, typically without meaningful optical consequences.^9^^,^^37^^,^^38^ Decentration exceeding ∼0.75 mm has been shown to cause noticeable optical deterioration in multifocal IOLs.^13^^,^^25^ In our study, mean decentration remained <0.25 mm, well below clinically relevant thresholds. Similar to decentration, optical quality—particularly MTF—is highly sensitive to even small degrees of IOL tilt.^11^^,^^38^^,^^39^ Although tilt was not quantitatively measured, no visible tilt was observed microscopically, and the consistently high MTF values indicate that no clinically meaningful tilt occurred. Taken together, these findings indicate a low risk of clinically significant visual impairment due to tilt or decentration when IOLs are fixated within the fixOflex device.

Postoperative capsular fibrosis and shrinkage remain major determinants of long-term IOL stability.^40^ Although several strategies aim to reduce PCO formation and capsular contraction—including optimized capsulorhexis, plate-haptic and sharp-edged IOL designs, and capsular tension rings—clinically meaningful decentration still occurs in a considerable proportion of patients.^41^^–^^43^ Newer concepts, such as femtosecond laser–assisted capsulotomies or capsulorhexis-fixating IOLs (e.g., FEMTIS), can improve centration, but their applicability is limited to specific surgical settings. Likewise, techniques such as the bag-in-the-lens design offer excellent centration and PCO prevention but require strict anatomic prerequisites and are therefore not widely adopted.^44^^–^^46^

The fixOflex device expands on these principles by mechanically reexpanding the capsular bag while providing integrated IOL fixation. This dual mechanism may counteract fibrotic contraction processes and support IOL stability in eyes with an elevated risk of postoperative capsular instability. In the present study, precise IOL positioning and centration within the device were evaluated in a standardized laboratory setting. In vivo, additional factors—such as variability in capsular bag size, zonular tension, and postoperative fibrotic processes of the capsular bag—will influence the implant's final position. Some of these aspects, such as zonular and capsular bag interactions during and after implantation of the fixOflex, are currently being investigated in dedicated experimental studies.

In addition, a potential clinical advantage of the fixOflex may be reversibility: because the IOL is clipped into the device rather than implanted directly in the capsular bag itself, the design may facilitate IOL exchange in complex cases where replacement is required.

Our study focused on the optical performance and centration of C-loop IOLs within the fixOflex under standardized laboratory conditions, enabling a direct and isolated investigation of the device's optical contribution. Optical bench testing allows the measurement of high-precision parameters—such as MTF, straylight, and centration—under controlled conditions that cannot be assessed with comparable accuracy in clinical settings. By using one-piece C-loop IOLs of identical material, design, and power, we minimized variability and confounding factors. The combined assessment of MTF, straylight, and decentration therefore provides a comprehensive characterization of the optical effects introduced by the fixOflex. This methodological approach, however, inherently limits generalizability. Future studies should include a broader range of IOL designs, including plate-haptic and four-point fixation configurations, as well as lenses with varying optic diameters, powers, and materials, to further validate the fixOflex concept. This investigation was conducted in collaboration with the inventor of the fixOflex device, I. G. Pallikaris, which may be viewed as a limitation. Nevertheless, the analysis and interpretation of the results were carried out independently, in accordance with the established standards of the David J. Apple Laboratory, and are supported by numerical data that are fully disclosed in the article.

## Conclusions

This laboratory study demonstrated that monofocal and multifocal one-piece C-loop IOLs maintained high optical quality when implanted into the fixOflex device, with no clinically meaningful increase in straylight and stable centration under bench conditions. These findings provide a quantitative basis for considering the fixOflex as a potential stabilizing platform for decentration-sensitive IOL designs, including aspheric, toric, and multifocal lenses. Clinical studies are required to confirm its safety, performance, and applicability in patient populations.

## Supplementary Material

Supplement 1

## References

[1] Keates RH, Pearce JL, Schneider RT. Clinical results of the multifocal lens. *J Cataract Refract Surg*. 1987; 13(5): 557–560. 3312575 10.1016/s0886-3350(87)80114-1

[2] Kelman CD . Phaco-emulsification and aspiration. A new technique of cataract removal. A preliminary report. *Am J Ophthalmol*. 1967; 64(1): 23–35. 6028631

[3] Neuhann T . Theory and surgical technic of capsulorhexis [in German]. *Klin Monatsbl Augenheilkd*. 1987; 190(6): 542–545. 3626415 10.1055/s-2008-1050454

[4] Nagy Z, Takacs A, Filkorn T, Sarayba M. Initial clinical evaluation of an intraocular femtosecond laser in cataract surgery. *J Refract Surg*. 2009; 25(12): 1053–1060. 20000286 10.3928/1081597X-20091117-04

[5] Donachie PHJ, Barnes BL, Olaitan M, Sparrow JM, Buchan JC. The Royal College of Ophthalmologists’ National Ophthalmology Database study of cataract surgery: Report 9, Risk factors for posterior capsule opacification. *Eye*. 2023; 37(8): 1633–1639. 36002508 10.1038/s41433-022-02204-1 PMC10219961

[6] Wei Z, Gordon P, Hao C, et al. Aged lens epithelial cells suppress proliferation and epithelial-mesenchymal transition-relevance for posterior capsule opacification. *Cells*. 2022; 11(13): 2001. 35805085 10.3390/cells11132001 PMC9265589

[7] Hartman M, Rauser M, Brucks M, Chalam KV. Evaluation of anterior capsular contraction syndrome after cataract surgery with commonly used intraocular lenses. *Clin Ophthalmol Auckl NZ*. 2018; 12: 1399–1403. 10.2147/OPTH.S172251 PMC6087024 30122893

[8] Meacock WR, Spalton DJ, Boyce J, Marshall J. The effect of posterior capsule opacification on visual function. *Invest Opthalmol Vis Sci*. 2003; 44(11): 4665. 10.1167/iovs.02-0634 14578383

[9] Eppig T, Scholz K, Löffler A, Messner A, Langenbucher A. Effect of decentration and tilt on the image quality of aspheric intraocular lens designs in a model eye. *J Cataract Refract Surg*. 2009; 35(6): 1091–1100. 19465297 10.1016/j.jcrs.2009.01.034

[10] Xu J, Zheng T, Lu Y. Effect of decentration on the optical quality of monofocal, extended depth of focus, and bifocal intraocular lenses. *J Refract Surg*. 2019; 35(8): 484–492. 31393986 10.3928/1081597X-20190708-02

[11] Chen XY, Wang YC, Zhao TY, Wang ZZ, Wang W. Tilt and decentration with various intraocular lenses: a narrative review. *World J Clin Cases*. 2022; 10(12): 3639–3646. 35647149 10.12998/wjcc.v10.i12.3639 PMC9100733

[12] Łabuz G, Auffarth GU, Yan W, Yildirim TM, Khoramnia R. Simulations of decentration and tilt of a supplementary sulcus-fixated intraocular lens in a polypseudophakic combination using ray-tracing software. *Photonics*. 2021; 8(8): 309.

[13] Soda M, Yaguchi S. Effect of decentration on the optical performance in multifocal intraocular lenses. *Ophthalmologica*. 2012; 227(4): 197–204. 22222365 10.1159/000333820

[14] Pallikaris IG, Elmassry A, Sahin O, et al. Intraocular lens stabilisation with a novel implant: one year follow up in a cohort of 120 patients. *Invest Ophthalmol Vis Sci*. 2022; 63(7): 2868–F0005.

[15] Pallikaris IG, Elmassry A, Ginis HS, et al. Safety and efficacy of a new endocapsular device used in age-related cataract surgery: twelve-month follow-up. *Transl Vis Sci Technol*. 2026; 15(2): 8. 10.1167/tvst.15.2.8 PMC12889166 41649457

[16] Ahmed E, Onurcan S, Loukia L, et al. Preservation of capsular transparency and geometrical consistency in cataract surgery using a novel intracapsular ring [published online November 9, 2020]. *J Clin Res Ophthalmol*. doi:10.17352/2455-1414.000082.

[17] Labuz G, Yildirim TM, van den Berg T, Khoramnia R, Auffarth GU. Assessment of straylight and the modulation transfer function of intraocular lenses with centrally localized opacification associated with the intraocular injection of gas. *J Cataract Refract Surg*. 2018; 44(5): 615–622. 29891155 10.1016/j.jcrs.2018.01.033

[18] Son HS, Labuz G, Khoramnia R, et al. Visualization of forward light scatter in opacified intraocular lenses and straylight assessment. *Diagn Basel*. 2021; 11(8): 1512. 10.3390/diagnostics11081512 PMC8393541 34441445

[19] Hammer M, Britz L, Schickhardt S, et al. Quantification of straylight induced by silicone oil adherent to intraocular lenses of different materials. *Am J Ophthalmol*. 2023; 262: 192–198. 38016528 10.1016/j.ajo.2023.11.018

[20] Britz L, Hammer M, Łabuz G, et al. Impact of calcium and phosphorus levels on optical deterioration in primary and secondary intraocular lens calcification. *Transl Vis Sci Technol*. 2024; 13(10): 18. 10.1167/tvst.13.10.18 PMC11472889 39388178

[21] Yildirim TM, Labuz G, Hammer M, et al. A novel approach for assessing visual impairment caused by intraocular lens opacification: high-resolution optical coherence tomography. *Am J Ophthalmol*. 2021; 226: 108–116. 33571474 10.1016/j.ajo.2021.02.001

[22] Labuz G, Vargas-Martin F, van den Berg TJ, Lopez-Gil N. Method for in vitro assessment of straylight from intraocular lenses. *Biomed Opt Express*. 2015; 6(11): 4457–4464. 26601008 10.1364/BOE.6.004457 PMC4646552

[23] van den Berg TJTP, Franssen L, Coppens JE. Performance of the C-Quant instrument for retinal straylight assessment. *Invest Ophthalmol Vis Sci*. 2006; 47(13): 1220. 10.1167/iovs.05-0690 16431978

[24] Guber I, Bachmann LM, Guber J, Bochmann F, Lange AP, Thiel MA. Reproducibility of straylight measurement by C-Quant for assessment of retinal straylight using the compensation comparison method. *Graefes Arch Clin Exp Ophthalmol*. 2011; 249(9): 1367–1371. 21567210 10.1007/s00417-011-1704-y

[25] Tandogan T, Son HS, Choi CY, Knorz MC, Auffarth GU, Khoramnia R. Laboratory evaluation of the influence of decentration and pupil size on the optical performance of a monofocal, bifocal, and trifocal intraocular lens. *J Refract Surg*. 2017; 33(12): 808–812. 29227508 10.3928/1081597X-20171004-02

[26] Mayer CS, Son HS, Łabuz G, et al. Laboratory and clinical experience with a diffractive trifocal intraocular lens sutured to an artificial iris. *J Refract Surg*. 2022; 38(1): 61–68. 35020535 10.3928/1081597X-20211209-02

[27] Lee Y, Łabuz G, Son HS, Yildirim TM, Khoramnia R, Auffarth GU. Assessment of the image quality of extended depth-of-focus intraocular lens models in polychromatic light. *J Cataract Refract Surg*. 2020; 46(1): 108–115. 32050240 10.1097/j.jcrs.0000000000000037

[28] Son HS, Tandogan T, Liebing S, et al. In vitro optical quality measurements of three intraocular lens models having identical platform. *BMC Ophthalmol*. 2017; 17(1): 108. 28662629 10.1186/s12886-017-0460-0 PMC5492950

[29] van Gaalen KW, Koopmans SA, Hooymans JMM, Jansonius NM, Kooijman AC. Straylight measurements in pseudophakic eyes with natural and dilated pupils: one-year follow-up. *J Cataract Refract Surg*. 2010; 36(6): 923–928. 20494762 10.1016/j.jcrs.2009.12.048

[30] van den Berg TJ, Franssen L, Kruijt B, Coppens JE. History of ocular straylight measurement: a review. *Z Med Phys*. 2013; 23(1): 6–20. 23182462 10.1016/j.zemedi.2012.10.009

[31] Van Den Berg T, Van Rijn LJR, Michael R, et al. Straylight effects with aging and lens extraction. *Am J Ophthalmol*. 2007; 144(3): 358–363. 17651678 10.1016/j.ajo.2007.05.037

[32] Łabuz G, Reus NJ, Van Den Berg T. Light scattering levels from intraocular lenses extracted from donor eyes. *J Cataract Refract Surg*. 2017; 43(9): 1207–1212. 28991619 10.1016/j.jcrs.2017.06.044

[33] Britz L, Schickhardt SK, Yildirim TM, Auffarth GU, Lieberwirth I, Khoramnia R. Development of a standardized in vitro model to reproduce hydrophilic acrylic intraocular lens calcification. *Sci Rep*. 2022; 12(1): 7685. 35538104 10.1038/s41598-022-11486-0 PMC9090772

[34] Britz L, Schickhardt S, Auffarth GU, Khoramnia R. Eintrübung hydrophiler Acrylintraokularlinsen: Überblick zu Labormethoden der histologischen Analyse und Replikation der Kalzifikation [Special issue]. *Klin Monbl Augenheilkd*. 2023; 240(8). 10.1055/a-2073-8526 37391183

[35] Britz L, Schickhardt SK, Yildirim TM, Auffarth GU, Lieberwirth I, Khoramnia R. Hydrophobic surface properties of hydrophilic acrylic lenses do not protect against calcification [in German]. *Ophtalmologie*. 2023; 120(10): 1022–1028. 10.1007/s00347-023-01862-0 37171476

[36] Mueller-Schotte S, van der Schouw YT, Schuurmans MJ. Ocular straylight: a determinant of quality of life in the elderly? *Gerontol Geriatr Med*. 2015; 1: 2333721415610193. 28138473 10.1177/2333721415610193 PMC5119907

[37] Xiao Z, Wang G, Zhen M, Zhao Z. Stability of intraocular lens with different haptic design: a swept-source optical coherence tomography study. *Front Med*. 2021; 8: 705873. 10.3389/fmed.2021.705873 PMC8455909 34568368

[38] Phillips P, Pérez-Emmanuelli J, Rosskothen HD, Koester CJ. Measurement of intraocular lens decentration and tilt in vivo. *J Cataract Refract Surg*. 1988; 14(2): 129–135. 3351748 10.1016/s0886-3350(88)80086-5

[39] Łabuz G, Yan W, Khoramnia R, Auffarth GU. Through-focus performance and off-axis effects in aspheric monofocal intraocular lenses. *Biomed Opt Express*. 2024; 15(10): 6073. 39421792 10.1364/BOE.533714 PMC11482183

[40] Tappin MJ, Larkin DFP. Factors leading to lens implant decentration and exchange. *Eye*. 2000; 14(5): 773–776. 11116703 10.1038/eye.2000.202

[41] Miller KM, Oetting TA, Tweeten JP, et al. Cataract in the adult eye preferred practice pattern. *Ophthalmology*. 2022; 129(1): P1–P126. 34780842 10.1016/j.ophtha.2021.10.006

[42] Ding X, Wang Q, Xiang L, Chang P, Huang S, Zhao YE. Three-dimensional assessments of intraocular lens stability with high-speed swept-source optical coherence tomography. *J Refract Surg Thorofare NJ 1995*. 2020; 36(6): 388–394. 10.3928/1081597X-20200420-01 32521026

[43] Nagata M, Hanemoto T, Matsushima H, Senoo T. Relationship between anterior capsule opening and direction of intraocular lens decentration. *J Cataract Refract Surg*. 2023; 49(9): 917–920. 37306397 10.1097/j.jcrs.0000000000001235

[44] De Keyzer K, Leysen I, Timmermans JP, Tassignon MJ. Lens epithelial cells in an in vitro capsular bag model: lens-in-the-bag versus bag-in-the-lens technique. *J Cataract Refract Surg*. 2008; 34(4): 687–695. 18361994 10.1016/j.jcrs.2007.11.055

[45] Tassignon M, De Groot V, Vrensen G. Bag-in-the-lens implantation of intraocular lenses. *J Cataract Refract Surg*. 2002; 28(7): 1182–1188. 12106726 10.1016/s0886-3350(02)01375-5

[46] Auffarth GU, Friedmann E, Breyer D, et al. Stability and visual outcomes of the capsulotomy-fixated FEMTIS-IOL after automated femtosecond laser-assisted anterior capsulotomy. *Am J Ophthalmol*. 2021; 225: 27–37. 33412122 10.1016/j.ajo.2020.12.025

