# Supplementary material for: Optical Quality and Centration Stability of a Novel Capsule Reconstructor for Intraocular Lens Fixation: Laboratory Evaluation of the fixOflex Device
Source: Transl Vis Sci Technol. 2026 Jul 6;15(7):5. doi: 10.1167/tvst.15.7.5 (PMC13349022; doi:10.1167/tvst.15.7.5)
Supplement: Supplement 1 [file tvst-15-7-5_s001.pdf]

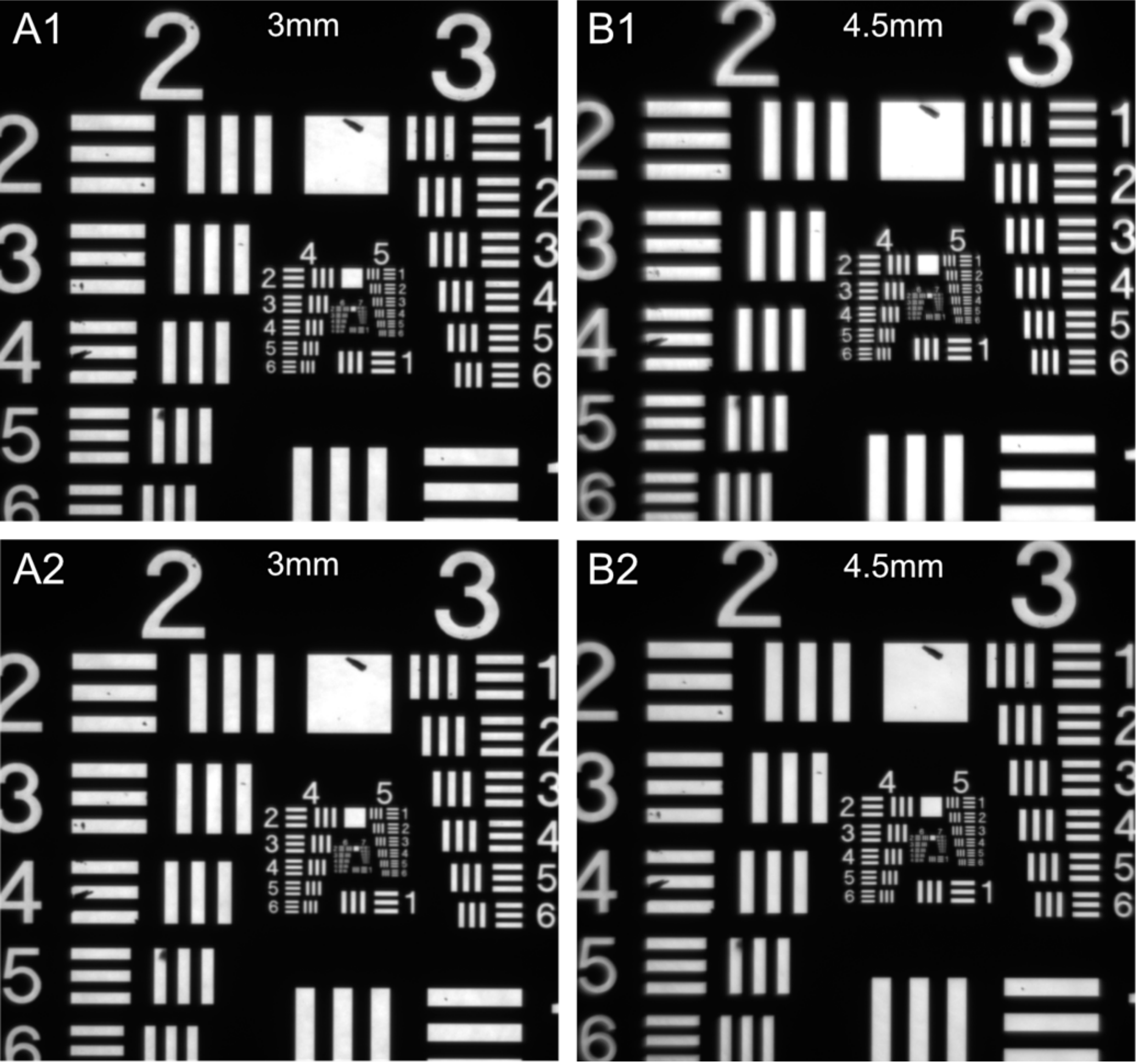

Supplementary Figure 1. Visualization of the changes in optical image quality for the monofocal intraocular lenses (IOLs) prior to (A1, B1) and after implantation into the fixOflex (A2, B2) at distance focus. The only noticeable difference in optical quality is attributed to the variation in aperture size, comparing 3 mm (A1, A2) and 4.5 mm (B1, B2).
